# Untangling the role of tau in sex hormone responsive cancers: lessons learnt from Alzheimer's disease

**DOI:** 10.1042/CS20230317

**Published:** 2024-10-29

**Authors:** Rachel M. Barker, Alfie Chambers, Patrick G. Kehoe, Edward Rowe, Claire M. Perks

**Affiliations:** 1 Cancer Endocrinology Group, Learning & Research Building, Southmead Hospital, Translational Health Sciences, Bristol Medical School, Bristol BS10 5NB, UK; 2 Department of Urology, Bristol Urological Institute, Southmead Hospital, Bristol BS10 5NB, UK; 3 Dementia Research Group, Learning & Research Building, Southmead Hospital, Translational Health Sciences, Bristol Medical School, Bristol BS10 5NB, UK

**Keywords:** Alzheimers disease, androgens, cancer, estrogens, PI3K/Akt signalling, tau proteins

## Abstract

Tubulin associated unit has been extensively studied in neurodegenerative diseases including Alzheimer's disease (AD), whereby its hyperphosphorylation and accumulation contributes to disease pathogenesis. Tau is abundantly expressed in the central nervous system but is also present in non-neuronal tissues and in tumours including sex hormone responsive cancers such as breast and prostate. Curiously, hormonal effects on tau also exist in an AD context from numerous studies on menopause, hormone replacement therapy, and androgen deprivation therapy. Despite sharing some risk factors, most importantly advancing age, there are numerous reports from population studies of, currently poorly explained inverse associations between cancer and Alzheimer's disease. We previously reviewed important components of the phosphoinositide-3-kinase/protein kinase B (PI3K/Akt) signalling pathway and their differential modulation in relation to the two diseases. Similarly, receptor tyrosine kinases, estrogen receptor and androgen receptor have all been implicated in the pathogenesis of both cancer and AD. In this review, we focus on tau and its effects in hormone responsive cancer in terms of development, progression, and treatment and in relation to sex hormones and PI3K/Akt signalling molecules including IRS-1, PTEN, Pin1, and p53.

## Introduction

Tau (tubulin associated unit) is more commonly known as a neuropathological intra-neuronal hallmark in neurodegenerative disease, most famously Alzheimer's disease (AD) and frontotemporal dementias, as well as chronic traumatic encephalopathy (CTE). Tau oligomerization and fibril formation, together with posttranslational modifications including hyperphosphorylation, are critical in the pathogenesis of such diseases, that are collectively described as tauopathies [1]. However, a role for tau in other pathologies has also been suggested, including non-neuronal cancers. Cancer and AD have similar risk factors, including advancing age, obesity, and insulin resistance, and the prevalence of both is increasing. However, an apparent inverse association appears to exist between the two diseases, such that the rates of co-occurrence of either disease in relation to the other is lower than would be expected, which is not attributable solely to survival bias [2,3]. An important role for differential regulation of the phosphoinositide-3-kinase (PI3K) signalling pathway, as one possible contributory mechanism that might explain this inverse association has been suggested previously [4].

An extensive literature on hormonal effects on tau in the brain highlights the importance of sex hormones in relation to risk and pathology of AD. This review explores beyond the brain and why tau may also play an important but unrecognized role in sex hormone driven cancers, such as breast and prostate.

## Tau overview

Tau, also termed MAPT (microtubule-associated protein) is encoded by *MAPT* on chromosome 17q21. Alternative splicing of exons 2, 3, and 10 on *MAPT* generate the six predominant tau isoforms observed in the adult human brain [5–7]. These are distinguished by a varying number of microtubule-binding repeats ‘R’ and N-terminal inserts ‘N’, denoted 0N/3R, 0N/4R, 1N/3R, 1N/4R, 2N/3R, and 2N/4R, and range between 45 and 65 kDa [7,8]. The expression of individual tau isoforms appears developmentally regulated, with only the smallest tau isoform, 0N/3R, observed during foetal development [9]. In the normal adult brain, the levels of 3R tau isoforms are approximately equal to that of the 4R isoforms, but this ratio is largely distorted in neurodegenerative disorders [10,11]. A number of pathogenic mutations observed within *tau* have been proposed to perturb the alternate splicing of exon 10, and imbalance the 1:1 isoform ratio in favour of 4R-containing tau isoforms [12].

Tau is primarily expressed in the central nervous system (CNS), especially neurons, although it has also been identified in astrocytes and oligodendrocytes in human and rat brains, respectively [13,14]. In a neuronal context, tau expression is enriched in axons and present in the synaptic and somato-dendritic compartments, including the plasma membrane, mitochondria, and nucleus [15–17]. Notwithstanding, tau expression has also been reported outside of the CNS, with the primary non-neuronal tissues in which measurable tau is observed including breast, prostate, kidney, gastric, and colorectal [https://www.proteinatlas.org/ENSG00000186868-MAPT/pathology] [18–22]

Unsurprisingly, the most well-established function of tau, is its capacity to bind and stabilize microtubules [23]. However, tau has since been recognized as a multi-functional protein playing a role in the mediation of neurogenesis, neuronal polarity, axonal stability, axonal transport, synaptic plasticity, synaptic function, cytoskeletal scaffolding, and DNA protection – dependent on its intracellular localization [24–27]. Attributing such diverse function, tau activity is intricately regulated at the transcriptional and post-translational levels; extensive tau post-translational modifications have been reported, including phosphorylation, acetylation, isomerization, oxidation, sumoylation, polyamination, and ubiquitylation [28,29].

**Figure 1:**
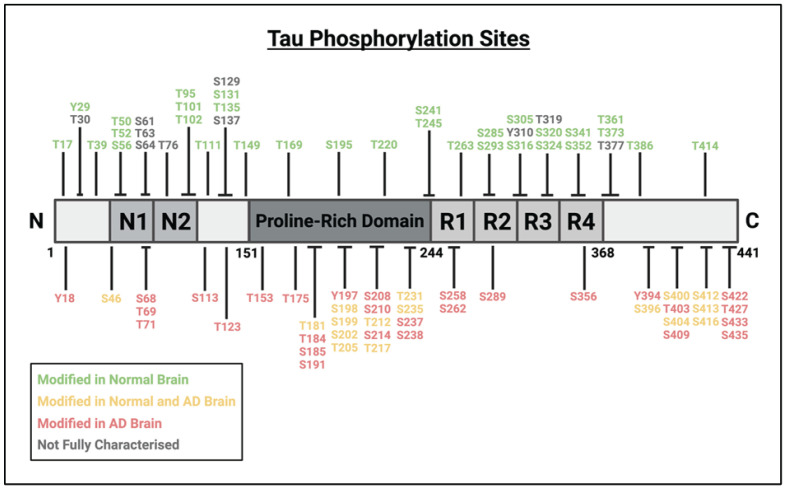
Putative phosphorylation sites mapped onto the sequence of the longest tau isoform, 2N/4R Putative phosphorylation sites mapped onto the sequence of the longest tau isoform, 2N/4R. Phosphorylation sites coded in green denote those observed as modified in normal adult brain, red in AD brain, yellow in both normal and AD brain, and grey for those that have not yet been fully characterized (produced using Biorender).

Amongst this array of modifications, perhaps the most pertinent to tau function is phosphorylation (Figure 1). Tau harbours a total of 85 putative phosphorylation sites along the largest tau isoform, with approximately 30 of these noted to regulate the normal function of tau, although the phosphorylation of additional sites are also observed during early development stages and in pathological contexts [30,31]. Interestingly, the phosphorylation of tau at various sites, differentially regulates its physiological function. For example, phosphorylation within the proline-rich domain inhibits its microtubule assembly activity, whereas phosphorylation within the C-terminal region increases this activity alongside its propensity for self-aggregation. There is a strong site-specificity to this, with tau phosphorylation at the Ser262, Thr231, and Ser235 residues reducing its microtubule-binding affinity by ∼35%, ∼25%, and ∼10%, respectively [32,33]. The phosphorylation of tau may also alter its proteasomal degradation and truncation *via* proteases. For instance, tau phosphorylation at Ser422 inhibits its caspase 3-mediated cleavage at Asp421 [34], while tau phosphorylation at either Ser262 or Ser356 prevents its recognition by HSP70-interacting protein-heat shock protein 90 (CHIP-HSP90), and thus its consequential degradation *via* the proteosome [35].

## Tauopathies

Once identified as the primary constituent of the neurofibrillary tangles seen in AD neuropathology, tau quickly became the subject of intensive research elucidating its role in the disease [36,37]. Following its hyperphosphorylation, tau protein dissociates from microtubules and self-aggregates into neurotoxic oligomeric structures [38]. These insoluble aggregates form intraneuronal inclusions of various morphologies and often gives rise to cytoskeletal destabilization, synaptic dysfunction, and neuronal cell death [39,40]. Collectively, these represent the hallmark neuropathological changes of the heterogenous group of neurodegenerative disorders appropriately termed tauopathies. Tauopathies are defined two-fold: primary tauopathies are diseases in which tau aggregates are considered the main factor associated with neurodegeneration, whilst secondary tauopathies involve additional pathogenic elements alongside tau aggregates [41]. Notable primary tauopathies include progressive supranuclear palsy (PSP), corticobasal degeneration (CBD), and Pick's disease, whereas Creutzfeldt–Jakob disease, CTE, and AD are considered secondary tauopathies [8]. Importantly, the neural inclusions underlying tauopathies also cause hyperphosphorylated tau protein to leak into the cerebrospinal fluid (CSF), detectable *via* enzyme-linked immunosorbent assay (ELISA) as a peripheral biomarker [39]. This often occurs prior to the emergence of initial clinical symptoms, enabling its use as a prognostic marker during early disease stages [39]. Considering its canonical role in neurodegenerative disorders, tau remains an attractive therapeutic target with many therapies, predominantly aimed at preventing its aberrant oligomerization, currently being tested at various phases of clinical trials [42].

## Tau in cancer

Expression data indicates that tau is present in many cancers, including sex-hormone-dependent tumours, such as breast, ovarian, and prostate (https://www.proteinatlas.org/ENSG00000186868-MAPT/pathology). Recently, a pan-cancer *in silico* analysis reported a significant association between *tau* expression and cell proliferation, inflammation, and epithelial-mesenchymal transition (EMT)-related genes [43], linking tau with established hallmarks of cancer [44]. This was supported by Clementi *et al* who demonstrated that mitotic phosphorylation of Tau modulates cell cycle progression in prostate cancer cells [45].

## Chemotherapy resistance

Taxanes are microtubule-targeting compounds commonly used as chemotherapy and work by causing cell cycle arrest and apoptosis. For example, key taxanes for breast cancer treatment include, paclitaxel and docetaxel and for prostate cancer, cabazitaxel [46]. Chemotherapy efficacy is limited by resistance and tau may be a mediator of resistance to taxane therapy by competing for tubulin binding sites [47]. Low tau expression has been associated with enhanced sensitivity to paclitaxel in breast [48–50], ovarian [51], gastric [52–56], and bladder cancer [57], and silencing tau expression in ZR75.1 breast cancer cells enhanced sensitivity to paclitaxel and docetaxel, but not epirubicin, doxorubicin, or vinorelbine [48,58], which are not taxanes, suggestive of a tau specificity toward tubulin-targeting drugs. Two compounds derived from benzoylphenylurea, that bind to the colchicine binding site on tubulin, used for treatment of pancreatic cancer also appeared to be more effective at inhibiting proliferation when tau levels were low [20]. Few tissue-based studies have been conducted in relation to chemoresistance and tau in prostate cancer, but one study showed no significant association between tau expression and docetaxel response [59]. However, *in vitro* studies have been more consistent with findings in other cancer types. For example, estramustine-resistant DU145 and docetaxel-resistant PC3 and DU145 prostate cancer cell lines showed higher levels of tau expression compared with their respective parental cell lines [60,61] and knocking down tau in PC3, and DU145 cells increased sensitivity to docetaxel in both parental and docetaxel-resistant cell lines, but the effect was more significant in the docetaxel-resistant cells [61]. However, one study showed that accumulation of tau oligomers following inhibition of autophagy increased sensitivity of PC3 and DU145 cells to docetaxel [62]. Whilst this review focusses on breast and prostate, it has also been noted that tau plays a role in chemoresistance in another hormone responsive tumour, high grade serous ovarian carcinoma [63].

## Tau and prognosis

*In silico* analyses have indicated that high expression of *tau* is associated with an increase in overall survival in patients with breast cancer, kidney clear cell carcinoma, and lung adenocarcinomas, but an inverse correlation has been reported for colon, head and neck cancer, and uterine cancer [22,43,64,65]. This cancer type specificity, with numerous context-dependent differences in relation to whether tau has prognostic value in terms of survival/treatment outcomes has been extensively reviewed previously [66]. The correlations of tau are also influenced by cancer-specific subtypes. For example, Lei *et al* reported that in triple negative breast cancer, tau expression was inversely associated with a pathological complete response. This association was not observed with human epidermal growth factor receptor-2 positive or negative subtypes [67]. Tau can also be measured in the serum and Darlix *et al* demonstrated that elevated levels of circulating tau represented an independent prognostic factor in these patients [68]. With prostate cancer, in contrast with breast, some studies reported that high tau expression in tumours is associated with a lower grade of cancer but with no association with the response to docetaxel [22,59,69]. A recent study reported statistically significant associations between prostate cancer risk and single nucleotide polymorphisms in *tau* [70]. There is evidence that tau influences prognosis by altering chemo-sensitivity and alters the efficacy of hormone cancer therapies. Low *tau* expression was associated with poor prognosis in estrogen receptor (ER)-positive breast cancers treated with tamoxifen [49]. Inhibiting tau production increased sensitivity of prostate cancer cells to the non-steroidal anti-androgen drug, bicalutamide and, in tissue, high *tau* expression was associated with poor overall survival in metastatic patients treated with androgen deprivation therapy (ADT) [69]. Androgen receptor (AR) synthesis was similarly reduced when tau was depleted and as bicalutamide resistance may be a consequence of AR overexpression [71], effects of tau on AR may contribute to its effects on hormone cancer therapy [69].

## Sex hormones and tau

The hypothalamic-pituitary-gonadal axis regulates gonadal hormone production and secretion, including progesterone, oestrogen, and androgen. The major sex hormones in women are progesterone and oestrogens, and in men, androgens. These have important functions in both men and women and are present to varying levels in both [72]. For example, Clark *et al* reported that in relation to testosterone levels, in healthy normal males, the lower end of the male range (8.8 nmol/L) was approximately four-fold higher than the upper end of the female range (2.0 nmol/L) [73]. Conversely, levels of oestrogen are much higher in ovulating females compared with males. Each of the sex hormones act on specific receptors. Progesterone interacts with the progesterone receptor, (PR) A and B, and oestrogen can act *via* two nuclear receptors, alpha and beta (ERα and ERβ), and a 7-transmembrane spanning G-protein-coupled receptor 1 (GPER1, also known as GPR30) and the effects of androgens are mediated by AR [74]. The main cancers in women influenced by oestrogens are breast, ovarian, and endometrial and for men, testosterone has significant effects on prostate cancer. Hence, hormone therapies have been designed to reduce systemic levels of these hormones or to block their actions on target tissues. Not surprisingly, sex-specific differences have been observed in many diseases, including cancer and AD [75].

The impact of tau on the efficacy of hormonal therapies is unclear and to gain some understanding of the role that tau may play in sex-hormone-dependent cancers, we can learn from assessing what is known about the impact of these hormones on tau in the brain. Evidence clearly shows that oestrogen and androgen regulate tau phosphorylation. For instance, loss of oestrogen increases the aging-related accumulation of phosphorylated tau in the rat hippocampus [76] consistent with neuroprotective actions of oestrogen. In AD, decreased expression of ER-α was associated with tau hyperphosphorylation and ‘pre-tangle’ formation [77]. Neuroprotective actions of progesterone [78] and androgens [79] have been similarly reported, with androgens reported to decrease tau phosphorylation in an AR-dependent manner [80]. Indeed oestrogen treatment attenuated tau hyperphosphorylation in mouse neuroblasts *in vitro* [81] and progesterone reduced tau expression in the rat cerebellum [82,83]. Both oestrogen and progesterone had similar effects on reducing tau phosphorylation *in vivo* in a transgenic mouse model of AD [84]. A further notable interaction between the ER and tau, was observed in the hippocampus and cortex of AD patient brains, in which neurofibrillary tangles (NFTs) with hyperphosphorylated tau were found to colocalize with ERα. This sequestration of ERα by tau inhibited the actions of oestrogen as demonstrated using the neuroblast M17 human cell line [85]. One study using both *in vitro* and *in vivo* mouse models determined that the two nuclear, ER receptors, α and β, had contrasting effects on tau phosphorylation: ERα positively, whereas ERβ negatively regulated tau phosphorylation [86]. This may suggest that the actions of oestrogen on tau are dependent on the context of ERs present in the tissue. Interestingly, oestrogen can affect cancer cells differently: promoting the growth of some such as those of the breast in which ERα is the main ER and inhibiting others, such as those of the colon in which ERβ predominates [87,88]. Notably, the relative importance of testosterone and oestrogen on tau pathology was demonstrated in AD mouse models, in which androgen depletion accelerated AD pathology, an effect prevented by androgen treatment. Testosterone may be metabolized to dihydrotestosterone (DHT) or estradiol (E2); mice treated with testosterone or E2 showed reduced tau phosphorylation whilst those treated with DHT showed no change, implying that androgen regulation of tau pathology largely occurs through oestrogen pathways [89].

To support the effects of these hormones on tau, associations with tau have also been reported in the brain following hormone replacement therapy (HRT) that replaces the female hormones, progesterone, and oestrogen, to alleviate the impact of the menopause, and hormonal cancer therapies. Early menopause, characterized by low oestrogen, has notably been associated with worse cognitive decline and pathology in AD, although trials of various HRT formulations as a therapy in AD have been inconsistent [90]. PET studies have shown enhanced tau deposition associated with early menopause and oophorectomy, and increased tau deposition in post-menopausal females compared with age-matched males [91–93]. Use of HRT, was associated with a statistically non-significant reduction in tau deposition [94], whilst late initiation of HRT resulted in increased tau [92]. Using a human neuroblastoma cell line (SH-SY5Y) and primary cultures of newborn male or female rat cerebral cortical neurons, oestrogen was shown to prevent tau hyperphosphorylation and induce dephosphorylation [95,96] and in female AD transgenic mouse models, hormone replacement following oophorectomy led to reduced tau phosphorylation [84].

**Figure 2:**
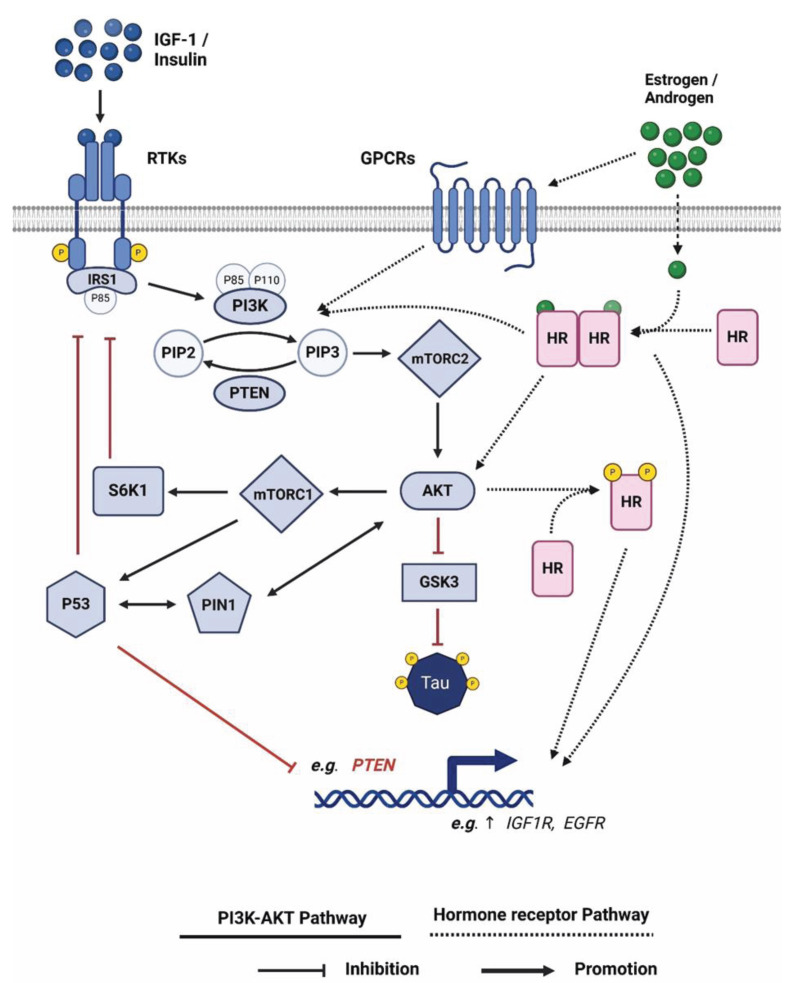
Schematic representation of the PI3K/Akt cellular pathway and its regulation by sex hormones This is a schematic of the PI3K/Akt cellular pathway, that depicts activation through ligand engagement of receptor tyrosine kinase receptors (RTKs) and sex hormone receptors, androgen, and oestrogen (AR and ER, respectively). Activation of the RTKs leads to tyrosine phosphorylation of insulin receptor substrate-1 (IRS-1) and activation of mammalian target of rapamycin complex 2 (mTORC2) and Akt. Activation of Akt inhibits GSK3 β that maintains a normal level of tubulin associated unit (tau) phosphorylation to bind and stabilize microtubules. Homeostasis is maintained partly by mTORC1 sensing of metabolic conditions, which leads to activation of tumour protein 53 (p53) and ribosomal protein S6 kinase beta-1 (S6K1) serine phosphorylation of IRS-1. In cancer, such negative feedback pathways are switched off leading to constitutive activation of Akt. Furthermore, peptidyl-prolyl isomerase (pin1) is upregulated and can further amplify Akt stability through serine 473 phosphorylation and by impairing the function of p53. Upregulation of Pin1 and inactivation of p53 switches the balance in favour of oncogenes (such as the insulin-like growth factor receptor [IGF-1R]) over tumour suppressors (such as phosphatase and tensin homolog [PTEN]) to promote tumorigenesis. The PI3K/Akt pathway can also be modified by sex hormones in both non-genomic and genomic ways. For example, oestrogen can phosphorylate and deactivate PTEN *via* G-protein coupled receptor 1 (GPER1) and androgens are able to increase levels of phosphoinositide-3-P phospholipids through a direct interaction of the AR and the p85alpha regulatory subunit of class I(A) PI3-K. Examples of genomic regulation include upregulation of the *IGF-1R* by androgens (produced using Biorender).

Whilst reduced oestrogen levels following menopause have been proposed as a risk factor for AD in women, use of oestrogen-modulating therapies for breast cancer treatment have been shown to have the opposite effect, being associated with decreased risk of AD [97,98]. HRT, while proposed by some to be protective against AD [90], increases risk of breast cancer, particularly ER-positive breast cancer [99], and the associations of oestrogen with reduced tau levels may offer further insight into this enhanced risk, but this is currently unknown. There is also the seemingly paradoxical complication, posed by the reported associations between high tau levels with better prognosis in some cancers although this may relate to different phosphorylation states of tau. Further evidence of a link between hormone therapy and tau comes from numerous studies that show an increased risk of AD in men who received ADT to treat prostate cancer [100–102]. Whilst there has been some inconsistency as some studies showed no such association [103,104] a recent meta-analysis of 28 studies confirmed an increased risk of AD following ADT [105]. The effects of ADT on amyloid β (Aβ) accumulation have been more studied than the effects on tau; however, in both mouse and rat pre-clinical models, depletion of endogenous testosterone was associated with increased phosphorylation of tau, an effect that was reversed by testosterone treatment [79,106]. In the mouse model, testosterone treatment led to a decrease in tau phosphorylation by downregulating glycogen synthase kinase 3 β (GSK3β) activity *via* protein kinase B (Akt) [79] (see Figure 2). A further study showed that reduced tau phosphorylation following hormone restoration occurred primarily as a result of oestrogen pathways as this effect was seen in mice treated with testosterone and oestrogen, but not with DHT [89].

## Sex hormones, tau, and PI3K signalling

As we described previously, the PI3K pathway is differentially modulated in cancer compared with AD, constitutively activated in cancer while inhibited in AD, contributing to conditions of cell over-growth and degeneration respectively [4]. The PI3K/Akt pathway is classically activated through stimulation of RTKs (including the insulin and insulin-like growth factor receptors [IR and IGF-1R, respectively]) [107–109] but can also be regulated through non-genomic and genomic actions of activated hormone receptors, including those for oestrogen and androgen (ER and AR, respectively) (Figure 2). In ovarian cancer, oestrogen upregulated the PI3K pathway in a non-genomic fashion through GPER, which resulted in the phosphorylation and inactivation of the tumour suppressor gene, the phosphatase and tensin homologue (PTEN) [110].

Androgens have also been shown to initiate transcription-independent effects that enhance PI3K signalling. In hormone responsive prostate epithelial cells, androgens acting *via* the AR increased levels of phosphoinositide phospholipids, that was associated with the phosphorylation of Akt/protein kinase B. Notably, these non-genomic actions of androgens required direct interaction between AR and the p85alpha regulatory subunit of class I(A) PI3-K [111]. Genomic regulation by oestrogen and androgen also plays a critical role in modulating the PI3K pathway. For example, oestrogen acting *via* ERα activated the PI3K pathway by genomically downregulating levels of PTEN [110]. In addition, in castration-resistant prostate cancers, androgens transcriptionally upregulate receptors for IGF-I and epidermal growth factor (EGF), that are key drivers of the PI3K signalling pathway [112] These effects culminate in crosstalk and feedback loops occurring between RTKs, androgen and estrogen signaling and the PI3K/Akt/mTOR signaling cascade [113,114]. For example, in PTEN-deficient prostate cancer, androgen-bound AR leads to an increase of Akt phosphorylation that in turn regulates AR transcriptional activity and expression [115].

There is limited knowledge as to a role of tau in PI3K signalling in cancer. Yet, drawing on existing knowledge of how key components of the PI3K pathway (insulin receptor substrate [IRS], PTEN, and peptidyl-prolyl isomerase [Pin1] and P53) and how these interact with tau in the brain, may provide insights.

IRS-1 is an important intracellular signalling intermediate of the IR and IGFIR, mediating effects *via* activation of the PI3K and the mitogen activated protein kinases (MAPK) pathway, that is parallel to the PI3K pathway downstream of RTKs [116]. Tyrosine phosphorylation of IRS-1 leads to downstream activation, whilst serine phosphorylation results in inhibition and leads to impaired insulin signalling [117]. Accordingly, tau knockout mice showed reduced tyrosine phosphorylation and increased serine phosphorylation of IRS-1, leading to insulin resistance in the brain, as well as effects on peripheral energy homeostasis and metabolism [118]. Aβ42 oligomers caused endoplasmic reticulum stress, leading to C-Jun N-terminal kinase (JNK) activation and serine phosphorylation of IRS-1 followed by tau hyperphosphorylation [119]. In the human AD brain, abnormal serine phosphorylation of IRS-1 was associated with tau pathology [120]. Notably, tau phosphorylation was not affected by knocking out IRS-1 in amyloid precursor protein (APP) transgenic mice, which suggested that IRS-2 is compensatory [121]. The role of IRS1 in cancer in relation to tau has not been examined in the same way. Yet, in cancer the PI3K signalling pathway is overactivated, associated with increased tyrosine and reduced serine phosphorylation of IRS1. It is therefore conceivable this would be associated with downregulation of GSK3β and inhibition of tau phosphorylation.

Tau is notably a key binding partner of PTEN in the normal brain *via* its proline-rich domain. Through this PTEN is inactivated to promote Akt phosphorylation and maintain neuronal health (Marciniak et al., 2017; Tai et al., 2020). A negative feedback loop is evident in AD whereby a reduction in Akt signalling, promotes GSK3β, causing hyperphosphorylated tau. (Hanger et al., 1992; Mandelkow et al., 1992). Hyperphosphorylated tau forms tangles and is no longer available to act as a break on PTEN, that further reduces Akt activation. Investigations into the role of tau in the regulation of PTEN in cancer are limited. However, associations between the two proteins have been observed in breast cancer. Human epidermal growth factor receptor 2 (HER2)-positive advanced breast cancer that demonstrated high tau-protein and low PTEN expression showed a significant association with poor response to chemotherapy [122].

Pin1 binds to and catalyzes the conversion of specific proline-directed serine/threonine phosphorylation motifs between *cis* and *trans* conformations of proline. This significantly alters the function of phosphorylated proteins, including that of tau [123,124]. Lu *et al* demonstrated that Pin1 binds tau in a cell-cycle-specific and phosphorylation-dependent fashion [125]. The important role that Pin1 plays in both neurodegenerative diseases and cancer is clearer and has been more extensively reviewed [126,127]. In AD, levels of Pin1 are reduced, thought to activate GSK3β to thereby cause hyperphosphorylation of tau and formation of NFTs. These NFTs then sequester Pin1 and disable its normal regulatory functions, making neurons more susceptible to cell death (Wang, Iqbal 2013). In contrast, in cancer, Pin1 is upregulated to promote tumour progression *via* activation of the Akt signalling pathway [reviewed in Zhou & Lu 2016), but its relationship to tau as a potential mediator or intermediary factor in this has not been assessed.

p53 is a transcription factor that plays an important role in response to DNA damage and maintenance of genome integrity. It is an effective tumour suppressor, regulating cell cycle, apoptosis, differentiation, and senescence [128]. In cancer, loss or mutation of p53 promotes activation of the PI3K pathway, whereas in AD, the opposite occurs, whereby p53 is upregulated and switches off the PI3K pathway [4]. A link between p53 and tau was observed in human embryonic kidney (HEK293) cells. This showed p53 enhanced phosphorylation of tau, although this appears to be an indirect effect as tau and p53 are predominantly expressed in different cellular compartments [129]. However, it has been reported that under pathological conditions, tau is not only located in the cytoplasm, but also in the nucleus, which may have implications for a role of tau in a range of cancer therapies, not just the taxanes that disrupt microtubules. Additionally, tau may mediate sensitivity to DNA damaging agents *via* its modification of p53 which would also have implications for cancer therapy [130–133].

## Summary

Increased reports of inverse associations between cancer and AD incidence present a seemingly contradictory phenomenon given the two conditions have similar risk factors. Regulation of the PI3K/Akt signalling pathway may be a vital pivoting mechanism to explain this inverse relationship as it is clearly differentially modulated in both diseases. The PI3K/Akt pathway is classically activated through stimulation of RTKs (such as the IR and IGF-1R) but can also be modulated by activation of hormone receptors (ER and AR). Some cancers are driven by sex-hormones, oestrogen, and testosterone, and in studies in women experiencing the menopause, HRT and ADT provide clear evidence of important hormonal effects in an AD context, particularly in relation to tau. In AD, dysregulation of PI3K/Akt signalling results in tau hyperphosphorylation, causing microtubule disassembly and the aggregation of free tau molecules. Learning about the hormonal regulation of tau and its role in neurodegenerative tauopathies, such as AD, may expand our current understanding of the role of tau in sex-hormone driven cancers and direct future research as to its potential contribution to upregulated PI3K signalling in cancer. This may lead to additional ways of effectively targeting this pathway and provide new options for other treatments and/or uncover new ways to reduce toxic side-effects of current treatments, optimize their efficacy in countering resistance and for those with advanced disease, providing means to extend their lifespan.

## Data Availability

Data sharing is not applicable to this review paper.

## Open Access

Open access for this article was enabled by the participation of University of Bristol in an all-inclusive *Read & Publish* agreement with Portland Press and the Biochemical Society under a transformative agreement with JISC.

## Abbreviations

AD: Alzheimer's disease
ADT: androgen deprivation therapy
AKT: protein kinase B
APP: amyloid precursor protein
AR: androgen receptor
Aβ: amyloid β
CNS: central nervous system
CTE: chronic traumatic encephalopathy
DHT: dihydrotestosterone
E2: estradiol
EMT: epithelial-mesenchymal transition
ER: estrogen receptor
GPER1: G-protein-coupled receptor 1
GSK3: glycogen synthase kinase 3
HRT: hormone replacement therapy
IGF1R: insulin-like growth factor receptor 1
IR: insulin receptor
MAPT: microtubule associated protein
NFTs: neurofibrillary tangles
PI3K: phosphoinositide-3-kinase
Pin1: peptidyl-prolyl isomerase
PR: progesterone receptor
PSA: prostate specific antigen
PTEN: phosphatase and tensin homolog
RFS: recurrence free survival
RTK: receptor tyrosine kinase
Tau: tubulin associated unit
